# MicroMap: a network visualisation resource for human microbiome metabolism

**DOI:** 10.1038/s41522-025-00853-0

**Published:** 2025-11-28

**Authors:** Cyrille C. Thinnes, Renee Waschkowitz, Eoghan Courtney, Eoghan Culligan, Katie Fahy, Ruby A. M. Ferrazza, Ciara Ferris, Angeline Lagali, Rebecca Lane, Colm Maye, Olivia Murphy, David Noone, Saoirse Ryan, Mihaela Bet, Maria C. Corr, Hannah Cummins, David Hackett, Ellen Healy, Nina Kulczycka, Niall Lang, Luke Madden, Lynne McHugh, Ivana Pyne, Ciara Varley, Niamh Harkin, Ronan Meade, Grace O’Donnell, Bram Nap, Filippo Martinelli, Almut Heinken, Ines Thiele

**Affiliations:** 1 Digital Metabolic Twin Centre, University of Galway, Galway, Ireland; 2 School of Medicine, University of Galway, Galway, Ireland; 3 Ryan Institute, University of Galway, Galway, Ireland; 4 APC Microbiome Ireland, Cork, Ireland; 5 University College Dublin, Dublin, Ireland; 6 University of Galway, Galway, Ireland; 7 University College Cork, Cork, Ireland; 8 Trinity College Dublin, Dublin, Ireland; 9 Inserm UMRS 1256 NGERE, University of Lorraine, Nancy, France; 10 School of Biological and Chemical Sciences, University of Galway, Galway, Ireland

**Keywords:** Microbiome, Microbial genetics, Biological techniques, Microbiology

## Abstract

The human microbiome critically influences metabolism and thereby our health. Constraint-based reconstruction and analysis (COBRA) is a proven framework for generating mechanism-derived hypotheses along the nutrition-host-microbiome-disease axis. However, no large-scale microbiome metabolism visualisation has been available. Therefore, we created the MicroMap, a manually curated network visualisation, which captures the metabolism of over a quarter million microbial genome-scale metabolic reconstructions. The MicroMap contains 5064 unique reactions and 3499 unique metabolites, including for 98 drugs. Users can intuitively explore microbiome metabolism, inspect metabolic capabilities, and visualise computational modelling results. Further, the MicroMap may serve as an educational tool to help diversify the computational modelling community. We generated 257,429 visualisations, covering all our current microbiome reconstructions, to visually compare metabolic capabilities between microbes. The MicroMap integrates with the Virtual Metabolic Human (VMH, www.vmh.life), the COBRA Toolbox (https://opencobra.github.io), and is freely accessible at the MicroMap dataverse (https://dataverse.harvard.edu/dataverse/micromap), along with all the generated reconstruction visualisations.

## Introduction

Human microbiome metabolism is intricately linked to health and disease^1^. Investigating the relationships between intrinsic (e.g., genetics) and extrinsic (e.g., nutrition) contributing factors requires a holistic approach that integrates multiple layers of biomedical information along the nutrition-host-microbiome-disease axis^2^. Computational approaches offer an attractive opportunity to tackle this dynamic and context-dependent systems biology challenge.

Genome-scale metabolic reconstructions (GEMs) are mathematical representations of an organism’s metabolism and enable the generation of mechanism-derived hypotheses in conjunction with the appropriate computational methods, such as constraint-based reconstruction and analysis (COBRA), *via*, e.g., the COBRA Toolbox^3^. Ongoing efforts enabled the continuous development of the requisite human and microbiome reconstructions^4^. Furthermore, the content of the human reconstructions Recon2^5^ and Recon3D^6^ was visualised as their metabolic network maps, ReconMap^7^ and ReconMap3^8^, respectively, to enable users to interactively explore human metabolism and display modelling and simulation results, such as predicted flux values using COBRA^9^. However, no comparable microbiome network visualisation counterparts have been available thus far.

Therefore, we created the MicroMap, a manually curated network visualisation of microbiome metabolism derived from the AGORA2 resource of 7302 human microbial strain-level metabolic reconstructions, which also captures the metabolism of 98 commonly prescribed drugs^10^. The MicroMap is also compatible with the metagenome-assembled genomes (MAG)-derived APOLLO resource of 247,092 microbial metabolic reconstructions^11^, as all reaction and metabolite identifiers are consistent throughout both resources, and APOLLO metabolic content, i.e., number of reactions and metabolites, is smaller than that of the reference genome-derived AGORA2 reconstructions. Users may explore the standalone MicroMap to inspect the systems context of microbiome metabolism, and integrate the MicroMap with their modelling methodologies, e.g., to visualise COBRA results.

By offering an accessible and interactive means to visually explore microbiome metabolism, the MicroMap extends the capabilities of the Virtual Metabolic Human database (VMH, www.vmh.life)^8^, complementing existing visualisation tools, such as the ReconMaps for human metabolism, which have provided value not only focusing on metabolism, e.g., by illustrating metabolic remodelling during COVID-19 infection^12^, but also through further integration with other biological systems, e.g., signalling networks for applications in cancer research^13^, and complementing an international community with focus on systems biology visualisations^14^. The MicroMap shall support research and education in human microbiome metabolism, providing critical insights into how these interactions influence health and disease.

## Results

### The MicroMap captures thousands of human microbiome metabolic reactions

**Fig. 1:**
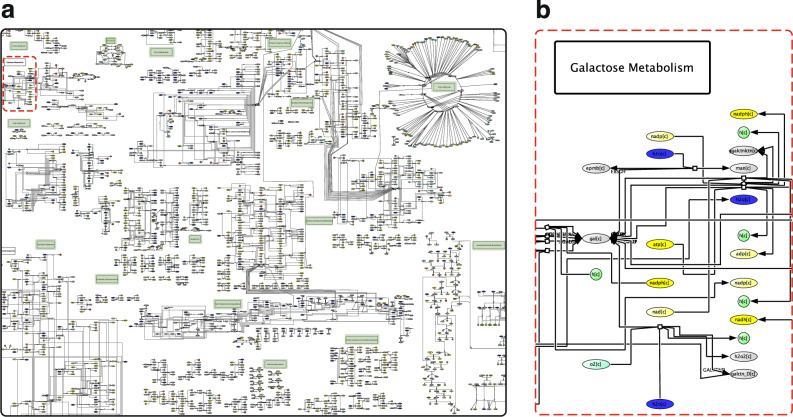
MicroMap overview. **a** A view of the MicroMap. Nodes represent metabolites and edges represent reactions. Highlighted in green are location labels, which indicate the names of metabolic subsystems or drugs to support intuitive map exploration. **b** A magnified view of the MicroMap location within the red dashed frame of (**a**). Metabolites contain their VMH identifier, with the associated compartment (e.g., c for cytosol) in brackets. We adopted a consistent colour scheme, e.g., by highlighting energy carriers in yellow, to support intuitive map exploration.

We created the MicroMap, which captures 5064 unique reactions (representing 59% and 76% of the AGORA2^10^ and APOLLO^11^ metabolic reconstruction resources, respectively) and 3499 unique metabolites (representing 97% and 100% of the AGORA2 and APOLLO resources, respectively), including the metabolism of 98 drugs (Fig. 1a). The city map-inspired design aims to intuitively guide the biochemically informed user, e.g., by clustering together related reactions, such as drug metabolism, and by providing 337 location signs, which indicate the relevant biochemical subsystem or drug name. Metabolites contain their VMH identifier, with the associated compartment (e.g., c for cytosol) in brackets. Reactions are labelled with their VMH identifier where feasible, i.e., not starting with a number (in which case they are displayed with their automatically assigned CellDesigner identifier). The use of VMH identifiers ensures that the MicroMap can be fully integrated both with the VMH and all its constituting resources, and with the COBRA Toolbox for modelling human-microbiome co-metabolism. We adopted a consistent colour scheme throughout, including similar hues for related molecules, e.g., yellow for energy carriers, such as nicotinamide adenine dinucleotide (nad[c]) or adenosine triphosphate (atp[c]) (Fig. 1b). The MicroMap CellDesigner .xml and .pdf files are freely available from the MicroMap dataverse (https://dataverse.harvard.edu/dataverse/micromap), alongside an onboarding tutorial (MicroMap Exploration with CellDesigner) and a worked case study (Scent Trails: tracking H_2_S production with the MicroMap).

The CellDesigner .xml files integrate with the COBRA Toolbox and can be opened in CellDesigner for browsing, searching, and editing. Please note that the image-derived .pdf version allows for quick inspection (e.g., in a browser), but cannot be searched.

### Reconstruction visualisations reveal differences in metabolic capabilities

**Fig. 2:**
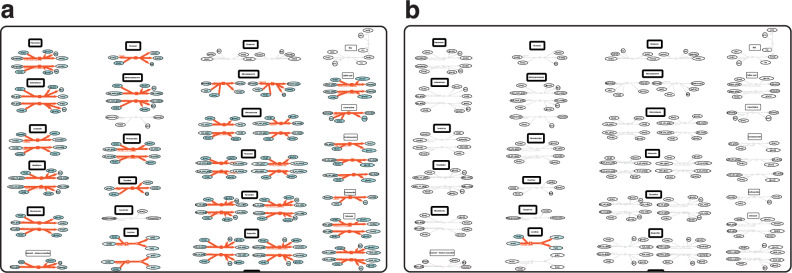
Comparison of microbial metabolic potential. **a** A view of the pan-Bacilli class reconstruction visualisation (red) on the MicroMap with focus on a drug metabolism section (same as in **b**). **b** A view of the pan-Verrucomicrobia class reconstruction visualisation (red) on the MicroMap with focus on a drug metabolism section (same as in **a**).

We then visualised microbiome reconstructions on the MicroMap to explore, compare, and contrast the known metabolic capabilities for different microbes. When comparing, e.g., the metabolic maps for Bacilli (Fig. 2a) and Verrucomicrobia (Fig. 2b), it is evident that Bacilli possess a relatively vast range of drug-metabolising capabilities compared to Verrucomicrobia. This visual means shall support making microbiome metabolism knowledge accessible to users, who may otherwise not be inclined to use biocomputational modelling software, e.g., in educational settings. We generated 9742 reconstruction visualisations for AGORA2, which includes visualisations for the 7302 strains, and for the pan-reconstructions, which include 39 classes, 78 orders, 152 families, 422 genera, and 1749 species. We also generated 247,687 visualisations for APOLLO, including for the 247,092 strains, and for the associated 595 pan-species reconstructions. The use of two high-performance high-memory servers enabled the parallel visualisation generation to complete within 22 days – a 40-fold enhancement over the use of a conventional desktop computer, estimated to have required 2.5 years of continuous visualisation generations. Altogether, we provide the full set of 257,429 microbe visualisations in CellDesigner .xml format, freely available from the MicroMap dataverse (https://dataverse.harvard.edu/dataverse/micromap).

### Heatmaps reveal relative reaction presence between microbes

**Fig. 3:**
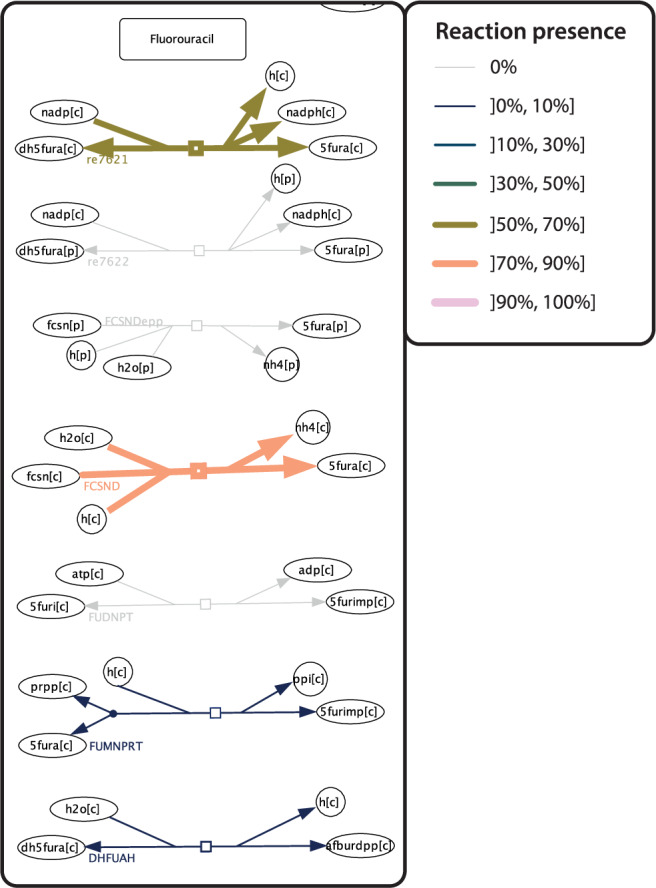
Heatmap of relative reaction presence on the MicroMap. A view of the relative reaction presence heatmap for 14 Pseudomonas species contained within AGORA2, with a focus on Fluorouracil drug metabolism. Both hue and line width are associated with relative reaction presence, ranging from fine-line navy blue to thick-line cotton candy. Light grey lines signify no reaction presence across the input reconstructions.

Further developing the capabilities for visual metabolism exploration, we implemented the capability to create heatmaps of relative reaction presence among a given set of reconstruction visualisations. We created, e.g., a heatmap, which represents the relative metabolic capabilities of 14 pseudomonas species contained within AGORA2 (Supplementary Fig. 1). Inspection revealed that the Pseudomonas subset can, e.g., mediate some known microbiome-mediated Fluorouracil^15^ reactions, with differing metabolic capabilities among the species (Fig. 3).

### Visualisations of metabolic modelling results reveal dynamic flux changes

**Fig. 4:**
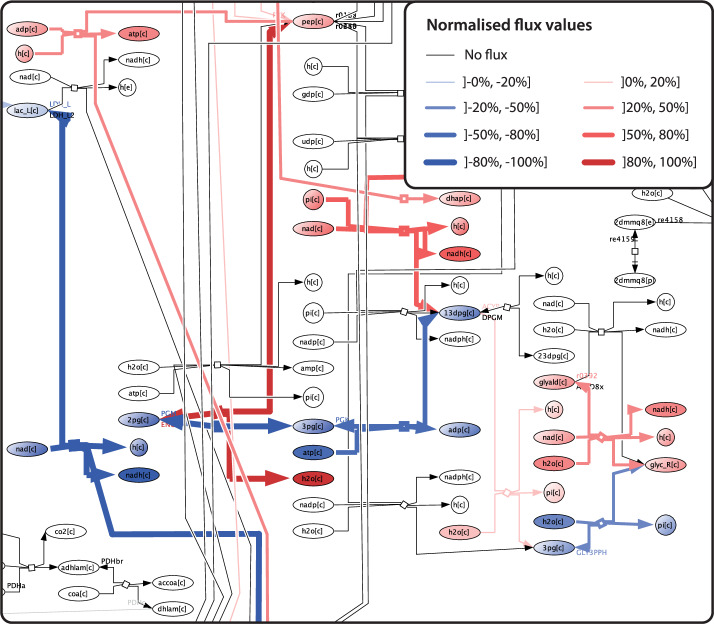
Visualisation of COBRA modelling results on the MicroMap. A view of an example flux vector visualisation on the MicroMap. Blue indicates negative flux values, red indicates positive flux values. Line width and hue reflect the magnitude of the reaction-associated flux. Grey lines signify that no flux is associated with the reaction.

We used the MicroMap to visualise a flux vector, which resulted from COBRA modelling and represents the flow of metabolites through a metabolic network (Fig. 4). By extension, we implemented the bulk visualisation of flux vectors issued from a longitudinal timeseries analysis. The resulting individual maps, representing one flux vector visualisation per time point, offered an opportunity to create a frame-by-frame animation to highlight flux dynamics over time. The animation revealed flux changes, including in sign and magnitude, e.g., for glutamate metabolism reaction P5CD (Supplementary Movie 1). Consequently, such animations are a means to visually identify potential candidate pathways of interest, based on their changes over time (or lack thereof). Please note, however, that a given flux vector may be part of multiple solutions for a set of linear equations – result interpretation shall therefore be informed by the specific COBRA methodology used.

### MicroMap visualisations enable mechanism-derived biochemical insights

By combining the above visualisation approaches, the MicroMap can be used to generate novel mechanistic insights into microbiome metabolism. The accompanying case study (Scent Trails: Tracking H₂S Production with the MicroMap) illustrates how the MicroMap enables users to contextualise specific metabolites or reactions within their broader network environment, including upstream precursors, downstream products, and associated biochemical subsystems. Inspection of the MicroMap, e.g., revealed that H₂S metabolism is largely captured by the sulphur, methionine/cysteine, and serine metabolism subsystems, with multiple direct and indirect routes for H₂S formation. We observed a recurring H₂S ↔ SO₃²⁻ interconversion motif, which interlinks organic and inorganic sulphur metabolism pathways. The MicroMap further provides a visual means to identify which microbes can (or cannot) participate in a given metabolic process. In our case study, we found that Desulfovibrio desulfuricans displays extensive H₂S metabolism, whereas Lactobacillus pasteurii shows none. Reaction presence heatmaps enable the user to visually assess relative metabolic capabilities across different taxonomic levels. We learned, e.g., that while some Lactobacillus species do have H_2_S-metabolising capabilities, most do not. Finally, the visualisation of flux balance analysis results can uncover differences in pathway usage under different conditions. The visualisation of flux results may reveal how alternative flux distributions can achieve the same metabolic objective through distinct routes, e.g., thereby highlighting pathway redundancies. In our case study, we observed that when no direct cysteine source was available, flux was routed through reactions synthesising cysteine, which suggests that Desulfovibrio desulfuricans produces cysteine endogenously under the given conditions. Together, these analyses demonstrate how the MicroMap facilitates hypothesis generation in a biochemical mechanism-informed manner.

## Discussion

The MicroMap is a manually curated metabolic network visualisation of the human microbiome. The MicroMap captures the metabolic content of over a quarter million microbial GEMs, encompassing ~5000 unique reactions and ~3500 unique metabolites, including those from 98 drugs. The MicroMap serves to 1. visually explore microbiome metabolism, 2. compare and contrast the metabolic capabilities of different microbes, and 3. visualise computational modelling results.

The MicroMap is fully integrated within the VMH and therefore seamlessly interconnects with all VMH resources, including human metabolism. The MicroMap complements the portfolio of biocomputational tools for studying human-microbiome interactions, such as the nutrition-gut-brain-health axis. The implemented visualisation methodologies, i.e., COBRA Toolbox functions, are transferable to related contexts, such as human metabolism-related visualisations involving the ReconMap3. Likewise, the already existing ‘Metabolic Cartography’ functions are compatible with microbiome visualisations involving the MicroMap.

While established databases like BRENDA^16^ or KEGG^17^ do provide metabolic network visualisations, those pathway maps encompass diverse taxa across the biosphere and are therefore not specific to the human microbiome. Further, they cannot be directly integrated with the VMH and COBRA modelling, e.g., for visualising flux results, because they do not interrelate with GEMs. MINERVA^18^ and Escher^19^ are effective tools for pathway visualisation in the COBRA context, but they rely on the availability of already existing maps, like the MicroMap for human microbiome metabolism. While there are several tools that create metabolic maps automatically from a GEM^20,21^, most remain only practical for small-scale networks, as the resulting maps will otherwise turn into ‘network hairballs’. The online tool Fluxer^22^ enables the rapid generation of GEM-derived networks and includes some modelling capabilities, yet the automatic layouts remain challenging to interpret in a biochemical context, with limited flexibility for custom COBRA modelling adaptations. We therefore deemed the manual creation of the MicroMap a necessary first step for enabling large-scale microbiome metabolic network visualisations in the COBRA context, which shall catalyse further refinement, integration, and collaboration with existing platforms, while building on the existing COBRA resources around the human ReconMaps.

Beyond its applications in research and development, the MicroMap also constitutes a potential educational tool for exploring, learning about, and communicating aspects of human microbiome metabolism. During the Virtuome summer school in digital health (https://www.digitalmetabolictwin.org/virtuome), undergraduate lectures, and presentations to non-expert audiences, e.g., the MicroMap has served as a catalyst to illustrate the systems aspects of microbiome metabolism, explore the associated scale and scope, and relate the reaction network content to genome-scale modelling. The visualisations aim to be accessible to users beyond computational metabolic modelling, including the wider scientific communities and stakeholders with a general interest in metabolism. After dabbling in generating heatmaps and animations, we encourage the user community to explore and propose new creative use cases for iterative improvement not only of the MicroMap, but also metabolic modelling visualisations in general.

## Methods

### Drawing the micromap

The MicroMap was created within the Virtuome summer school in digital health (https://www.digitalmetabolictwin.org/virtuome), based on the three pillars of biocomputational research, personal development, and community-engaged research. Virtuome participants were current STEM undergraduates in Ireland, including those from the University of Galway, University College Cork, University College Dublin, and Trinity College Dublin. The team was dispersed across Ireland and collaborated fully online, according to best practices in systematic team empowerment^23^.

At the start, we used MATLAB to export the biochemical subsystems, unique reactions, and metabolites from the AGORA2 resource as Excel spreadsheets. We then clustered the reactions according to their biochemical subsystem, as already assigned within the AGORA2 resource, which helped us structure the rather large manual drawing task into smaller tractable segments and also provided an estimate of the required area on the map, informed by the number of reactions.

We manually drew the metabolic reactions using CellDesigner (https://celldesigner.org)^24^. Considering the large number of 8,635 AGORA2 reactions to be completed within the limited 14-week part-time Virtuome component, we decided to prioritise biotransformation reactions and deprioritise exchange and transport reactions, i.e., the MicroMap contains all AGORA2 biotransformation reactions but only a small subset of exchange and transport reactions. Therefore, while exchange and transport reactions remain part of the overall modelling pipeline, e.g., the Microbiome Modelling Toolbox 2.0^25^, which uses full GEMs, they will not be visualised on the MicroMap. However, users may choose to add specific reactions to the MicroMap in CellDesigner if required for their use case.

We annotated the MicroMap with labels referring to the biological subsystems contained within AGORA2 (a list of which is available on the MicroMap dataverse), also including drug names, to help the user find the reactions of interest. We used colours to convey visual cohesion, e.g., by using the same colour for the same metabolites, and related colour schemes for related metabolites, such as shades of yellow for energy carriers. To facilitate efficient and consistent mapping, we established a template for recurring reaction layouts, metabolites, and associated colour schemes, which enabled us to copy and paste the ready-made constituting parts into the map in progress. After each contributor had created a handful of submaps, they integrated them into an iteratively growing, new version of the MicroMap.

Throughout, we aimed to adopt a city map-inspired design organised around subsystem nodes to facilitate intuitive navigation by the biochemically informed user. We guided the map integration process by encouraging each contributor to continuously consider the three aspects of content, design, and quality control. Content focused on the accurate representation of the parent AGORA2 reconstruction resource, design on creating a visually coherent layout, and quality control on ensuring good practice for minimising human error during the mapping process.

Once we completed a first manually assembled MicroMap draft, we used the COBRA Toolbox Metabolic Cartography functions, notably *checkCDerrors.m*, to compare the MicroMap with the AGORA2 resource, which highlighted any reaction and metabolite differences, including identifiers and reaction reversibility. We iteratively corrected the map such that all reactions and metabolites of the MicroMap are present within AGORA2. The most common mistake was the accidental introduction of a space at the beginning or end of an identifier, which are particularly challenging to spot with the naked eye. We then accounted for the unique metabolite content, i.e., metabolites with the same VMH identifier without the associated compartment, by creating the *uniqueMetabolites.m* and *uniqueSpeciesInMap.m* functions, which count unique metabolites in the AGORA2 model and those represented in the MicroMap, respectively.

### Visualising reconstructions

To visualise individual microbial reconstructions on the MicroMap, we created the *visualizeReconstructionsOnMap.m* COBRA Toolbox function, which visualises the reaction presence from each provided reconstruction onto the MicroMap. The function takes as input a CellDesigner .xml file and a folder path containing the reconstruction .mat files of interest. The strain-level AGORA2 and APOLLO metabolic reconstruction files (in .mat format) were downloaded from the VMH (www.vmh.life) and the Harvard Dataverse (10.7910/DVN/RL7E7G), respectively. The AGORA2 class-, order-, family-, genus-, and species-level, and the APOLLO species-level pan reconstructions were generated using the *createPanModels.m* Microbiome Modelling Toolbox 2.0 function, which merges individual reconstructions belonging to the same taxon into a single pan-model^25^. For the bulk reconstruction visualisations, we used high-performance Dell PowerEdge 730 servers, with 40 CPU cores and 768 Gb of RAM.

### Creating reaction presence heatmaps

To generate heatmaps of reactions present in a given set of microbial reconstruction visualisations, we created the *visualizeNormalizedRxnPresence.m* COBRA Toolbox function, which visualises relative reaction presence across multiple CellDesigner .xml maps. The function takes as input a folder path containing the reconstruction visualisation .xml files of interest.

### Visualising flux vectors

We computed a flux vector using flux balance analysis (*optimizeCBmodel.m*) using the COBRA Toolbox. The flux vector was then stored in .csv format, which contained reaction identifiers in the first column and associated, predicted flux values in the second column. To visualise the flux vector, we created the *addFluxFromFileWidthAndColor.m* and *visualizeFluxFromFile.m* COBRA Toolbox functions. The former applies flux magnitude and direction to a parsed metabolic map using reaction line width and colour, the latter applies this visualisation to a given CellDesigner .xml file and saves the resulting map. Inputs are a CellDesigner .xml file and a flux vector .csv or .xlsx file.

### Animating flux visualisations

We obtained an example flux vector series, issued from a longitudinal series of timepoints in .xlsx format. To visualise each flux, we created the *addFluxWidthAndColor.m* and *visualizeFluxTimeseriesFromFile.m* COBRA Toolbox functions. The former assigns flux magnitude and direction to a parsed metabolic map *via* reaction line width and colour, the latter applies this visualisation across all timepoints in a flux timeseries and saves each map as a separate CellDesigner .xml file. Inputs are a CellDesigner .xml file and an .xlsx file containing a series of flux vectors.

To generate a flux animation (Supplementary Movie 1), we exported each CellDesigner .xml file as a .pdf and imported them as layers in Adobe Photoshop. We then cropped to the region of interest, created a frame animation with 0.25 s delay, which we exported as a movie file.

## Supplementary information

Supplementary information

Supplementary Movie 1

## Data Availability

The MicroMap and all the created AGORA2 and APOLLO reconstruction visualisations are freely accessible and can be downloaded from the MicroMap dataverse (https://dataverse.harvard.edu/dataverse/micromap), in addition to an onboarding tutorial (MicroMap Exploration with CellDesigner) and a worked case study (Scent Trails: tracking H_2_S production with the MicroMap). The CellDesigner .xml files can be viewed, explored, and further edited using the open-access CellDesigner software (https://celldesigner.org). The created MATLAB functions are available from within the COBRA Toolbox (https://opencobra.github.io).

## Code availability

The created MATLAB functions are available from within the COBRA Toolbox (https://opencobra.github.io).
