# Supplementary material for: MicroMap: a network visualisation resource for human microbiome metabolism
Source: NPJ Biofilms Microbiomes. 2025 Nov 28;11:222. doi: 10.1038/s41522-025-00853-0 (PMC12663093; doi:10.1038/s41522-025-00853-0)
Supplement: Supplementary file 1 — Supplementary information [file 41522_2025_853_MOESM1_ESM.pdf]

Supplementary Figure 1

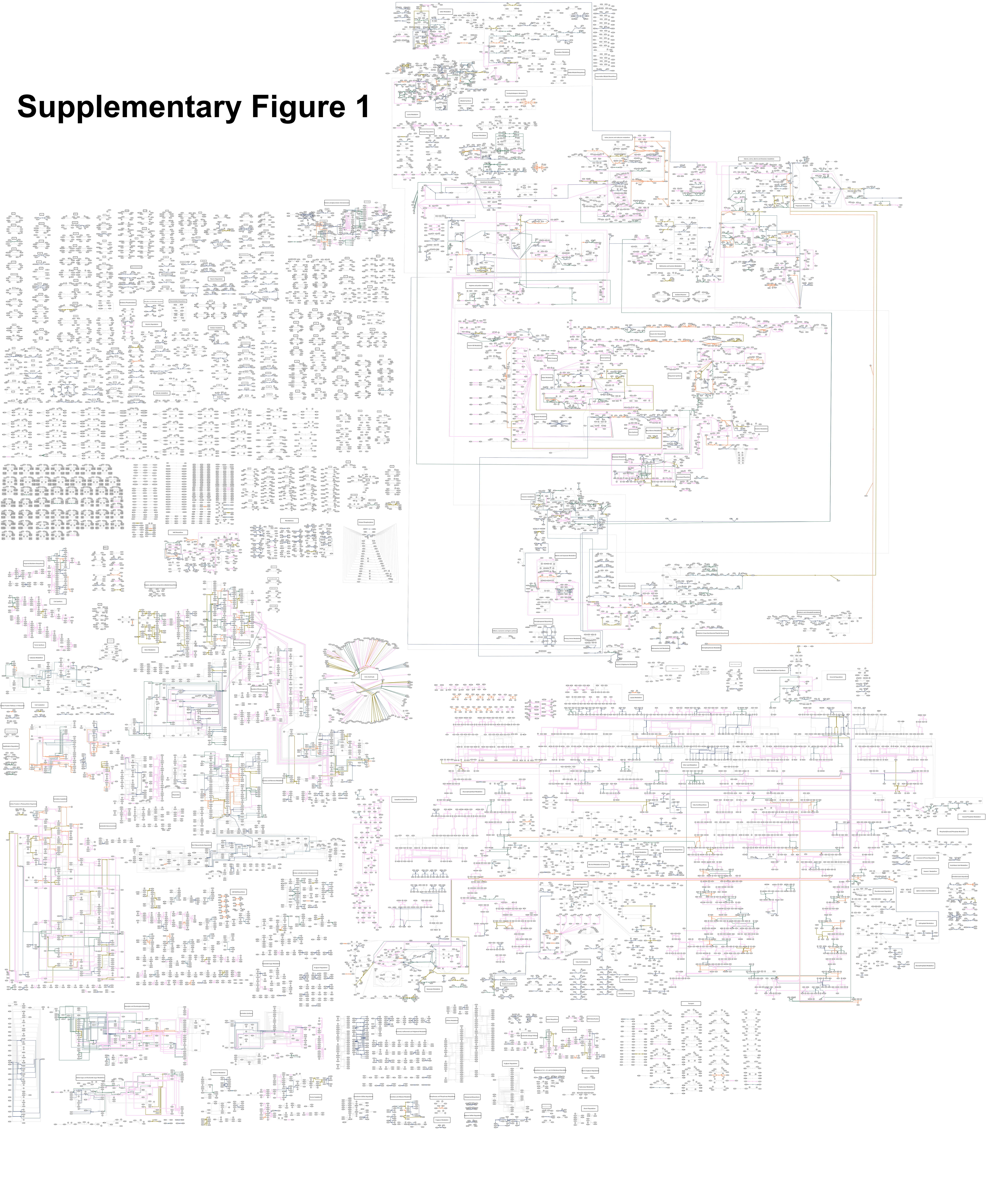

Supplementary file legends

Supplementary Figure 1

Heatmap of relative reaction presence on the MicroMap. Relative reaction presence heatmap for 14 Pseudomonas species contained within AGORA2. Both hue and line width are associated with relative reaction presence, ranging from saturated blue with fine lines to saturated red with thick lines. Grey lines signify no reaction presence across the input reconstructions. For legend details, please refer to **Figure 3** in the main manuscript.

Supplementary Movie 1

Animation of flux dynamics visualised on the MicroMap across a longitudinal time series. Each frame represents a flux vector at one timepoint. Line width and colour indicate flux magnitude and direction. For legend details, please refer to **Figure 4** in the main manuscript.
